# Comprehensive Travel Health Education for Tour Guides: Protocol for an Exploratory Sequential Mixed Methods Research

**DOI:** 10.2196/33840

**Published:** 2022-05-23

**Authors:** Ni Made Sri Nopiyani, Pande Putu Januraga, I Md Ady Wirawan, I Made Bakta

**Affiliations:** 1 Doctoral Program of Medical Sciences Faculty of Medicine Udayana University Denpasar Indonesia; 2 Department of Public Health and Preventive Medicine Faculty of Medicine Udayana University Denpasar Indonesia

**Keywords:** travel health, health education, tour guides, tourists, health promotion

## Abstract

**Background:**

Tourists are at risk of experiencing health problems during their travel. However, even though tour guides have the potential to become travel health promoters, their participation has not been optimal.

**Objective:**

This study aims to develop a comprehensive travel health education model to help tour guides improve health information delivery to tourists.

**Methods:**

This is an exploratory sequential mixed methods research. The first phase consisted of a qualitative study with an informed grounded theory design. In-depth interviews were carried out with tour guides from all language divisions and policymakers of the Indonesian Tour Guide Association Bali Branch or Himpunan Pramuwisata Indonesia Daerah Bali (HPI Bali). The interview guidelines were developed based on the theory of planned behavior and identity theory. Qualitative data were analyzed thematically. In the interim phase, a travel health education model and questionnaire were developed based on the qualitative findings. The initial model and its instruments were finetuned after consultation with travel medicine and health promotion experts. Furthermore, the validity and reliability of the questionnaire were tested on 30 tour guides. The second phase consisted of a quantitative study with a randomized pretest-posttest control group design. A total of 76 tour guides in the intervention group received comprehensive travel health education, while 76 in the control group received no specific intervention. Outcome variables (ie, attitudes, subjective norms, perceived behavioral control, actual behavioral control, role identity, and behavioral intention) were measured at baseline (T_0_), after the online training (T_1_), before information sharing via WhatsApp (T_2_), a month after the start of the WhatsApp intervention (T_3_), and at the end of the WhatsApp intervention (T_4_). The mean difference of each outcome variable before and after the intervention will be compared between the intervention and control groups. Thereafter, the quantitative and qualitative findings will be integrated into a joint display.

**Results:**

The qualitative phase was conducted through in-depth interviews with 21 informants who included tour guides and policymakers from HPI Bali from May to June 2021. The education model, educational materials, and questionnaire were developed based on the qualitative findings and consultation with experts. The education model consists of online training and information sharing through WhatsApp and was trialed with tour guides from November 2021 to February 2022. As of April 2022, this study is in the quantitative data analysis stage.

**Conclusions:**

A travel health education model was developed based on qualitative findings and consultation with experts. The model was tested with tour guides, and a series of self-administered questionnaires were completed. This study is in the quantitative data analysis stage and will continue by integrating qualitative and quantitative findings into a joint display.

**Trial Registration:**

ClinicalTrials.gov NCT04961983; https://clinicaltrials.gov/ct2/show/NCT04961983

## Introduction

### Background

Tourists are an epidemiologically important population because they are potentially exposed to diseases outside their home country and may serve as a conduit for diseases from their origin to destination country or vice versa [1]. Tourists visiting tropical, subtropical, and developing countries, including Indonesia, are at a higher risk of morbidities and mortalities due to the lack of hygiene, sanitation, safety, and disease endemicity of the destinations [2-5].

Several studies in Australia revealed less than optimal pretravel health behavior of tourists visiting Southeast Asia, including Indonesia. There were low proportions of tourists seeking pretravel consultation, receiving malaria chemoprophylaxis, and receiving a vaccination for typhoid and hepatitis B [6-8]. According to the literature, health problems that tourists experience during their visits to Indonesia include diarrhea, tropical infections (eg, dengue hemorrhagic fever, typhoid, malaria), respiratory infections (including COVID-19), animal bites (ie, rabies), methanol intoxication, and injuries or trauma due to accidents in tourism sites or traffic accidents [3,9-17]. Mapping of safety hazards and risks based on the World Health Organization (WHO) criteria for 197 tourist attractions in Bali concludes that 6.6% are high-risk areas, 39.1% are moderate risk, and the rest are low risk [14,15].

These facts indicate that tourists need local, specific, and up-to-date information on the prevention of travel-related health problems to adopt appropriate prevention measures. A tour guide is one actor in the tourism industry that potentially delivers health information to tourists due to their intensive contact with tourists during their visits [18-20]. As one of the best-known tourism destinations in the world, Bali is a province with the highest number of international tourist visits in Indonesia [21]. Bali has a great potential to involve tour guides in travel health promotion because it boasts 5470 registered tour guides, which is more than half of the total number of tour guides in Indonesia [22]. However, a cross-sectional study in Bali suggested that about half of the tour guides in Bali never or rarely provide health information to the tourists they serve [23]. This indicates the need for improved tour guide behavior and a need to address its determinants, namely attitude, subjective norms, role identity, self-efficacy, and knowledge [23].

There is no published literature to date on interventions to improve tour guides’ involvement in travel health promotion. Therefore, a comprehensive, relevant, and effective intervention addressing determinants of tour guides’ behavior in providing travel health information should be developed. Accordingly, this study aims to develop a comprehensive travel health education intervention relevant to the needs of tour guides providing travel health information to tourists in Bali. In addition, this study aims to assess the efficacy of the intervention to improve tour guides’ attitude, subjective norms, perceived behavioral control, actual behavior control, role identity, and intention to provide information regarding the prevention of travel health problems.

### Theoretical Foundation

An integration of the theory of planned behavior (TPB) and identity theory was employed as the reference for developing the intervention because the constructs of these theories are relevant to the determinants of tour guides’ behavior in travel health promotion [23-26]. TPB purports that human behavior is determined by three types of considerations: behavioral beliefs, normative beliefs, and control beliefs. Each of these beliefs will shape attitudes toward behavior, subjective norms, and perceived behavioral control, which in turn will affect behavioral intention. Behavioral intention and actual behavior control will ultimately determine the implementation of a particular behavior [26,27].

TPB has been used extensively in the development of behavior change interventions in the last three decades, including in the health sector [28-30]. TPB is used in this study because the delivery of travel health information by tour guides is a planned and voluntary behavior influenced by both internal and external determinants. There are limited publications related to the application of TPB in interventions to change actors’ individual behavior to become health promoters with the primary purpose of changing the health behavior of others [29,30]. The application of TPB to interventions that target nonhealth actors to make them health promoters in the context of travel health is even less explored, calling for further study [31].

A tour guide’s role as a nonhealth actor is a determinant that can influence their health-promoting behavior. Therefore, this research proposes an extended TPB by integrating identity theory into TPB [28]. The travel health education model developed in this study is a comprehensive intervention as it targets all determinants of health behavior based on the construct of extended TPB.

## Methods

### Trial Registration

This study was registered in ClinicalTrials.gov (NCT04961983).

### Study Design

**Figure 1:**
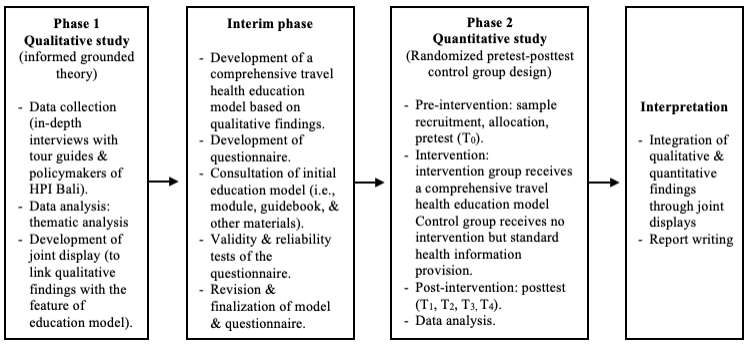
Workflow of an exploratory sequential mixed methods study to develop a travel health education model.

This research employs an exploratory sequential mixed methods design, in which the collection and analysis of qualitative data are conducted prior to the quantitative data [32,33]. The qualitative study results in the first phase became the basis for the intervention development in the interim phase. The intervention was then tested for its efficacy in the quantitative study. The workflow is presented in Figure 1.

### Study Setting and Population

This study was conducted in Bali, Indonesia. The study population comprised of 5470 tour guides who are registered members of the Indonesian Tour Guide Association Bali Branch or Himpunan Pramuwisata Indonesia Daerah Bali (HPI Bali).

### Phase 1: Qualitative Study

#### Study Design

The first phase of this research consisted of a qualitative study with an informed grounded theory, a design in which processes and products are developed in accordance with grounded theory but informed by existing theoretical frameworks [34].

#### Informants

At the beginning of this study, purposive sampling was employed, a nonrandom method ensuring that particular categories of informants within the population were represented in the study [35]. Informants were recruited based on the category of language division. There are 11 language divisions in HPI Bali: English, Japanese, Korean, Mandarin, France, Dutch, Italian, Spanish, Russian, German, and Indonesian. Every language division in HPI Bali has its own market countries (eg, tour guides in the English language division serve tourists from English-speaking countries). Therefore, 11 tour guides from all language divisions of HPI Bali were interviewed to gain a comprehensive understanding of the health education needs of tour guides serving tourists from various countries. In addition to the tour guides, we conducted in-depth interviews with 2 policymakers from HPI Bali to explore more perspectives regarding a feasible and relevant travel health education model to address tour guides' needs. Based on the ongoing data analysis results, 8 tour guide informants were recruited using theoretical sampling, recruitment based on data that require further exploration [35]. These informants were recruited through HPI Bali or snowballed from the previously interviewed informants. The prospective informants received an explanation of the research, and those who were willing to participate were asked to provide written consent.

#### Interview Guides

Questions for the semistructured interview guide were developed with reference to the constructs of the TPB, which was integrated with the identity theory. The interviews explored the following themes:

- Health problems experienced by tourists during their visit
- Attitude, subjective norms, perceived behavior control, role identity, actual behavioral control, and intention to provide information regarding travel health
- Provision of travel health information to tourists and barriers faced in conveying the information
- Tour guides’ needs for travel health information
- Educational strategies pertinent to tour guides’ needs (content, mode of delivery, duration, setting, supporting facilities)

All topics were explored through open questions. The order of questions was flexible according to each informant's response during interviews. Other information outside the interview guide that was interesting and important was also explored by the interviewers.

#### Data Collection

Data collection was carried out in person following COVID-19 health measures (ie, wearing masks and physical distancing) in the place and time agreed by the informant and interviewer. Interviews were audio recorded with the informants’ consent. Data collection and analysis were conducted concurrently. Recruitment of informants were stopped when the data reach theoretical saturation, the point at which no new theoretical insight emerges [35].

#### Data Analysis

The qualitative data were analyzed using inductive thematic analysis, in which the researcher identifies patterned themes [36]. Thematic analysis steps include interview transcription, familiarization of data, open coding of the entire data, axial coding (identifying the association of codes and integrating associated codes into a thematic category), selective coding (selecting and integrating categories into main themes to construct a theory), and report writing. The report also contained a joint display to link the qualitative findings with the detailed components of intervention design. Strategies to increase the trustworthiness of the research findings included triangulation of sources and peer debriefing with experts in travel health and health promotion [33]*.* NVivo 12 Plus software was utilized in the data analysis process.

### Interim Phase

In the interim phase, the qualitative findings were used to develop an intervention in the form of a travel health education model and its instruments (ie, travel health promotion guidebook for tour guides, training module, and media for WhatsApp information sharing), as well as a questionnaire to be tested in the quantitative study. The integrative procedure in this study was represented in a joint display. Joint display explicitly presents how the codes and themes that emerged in qualitative research have shaped the intervention elements [32]. The initial education model, its instruments, and the questionnaire were refined after consultation with travel medicine, health promotion, and tour guiding experts who provided input regarding the content and delivery formats. The questionnaire then underwent a pilot test on 30 tour guides. The validity and reliability of the questionnaire were analyzed, and revisions were made accordingly.

### Phase 2: Quantitative Study

#### Study Design

The research then continued with a quantitative strand using an experimental randomized pretest-posttest control group design. The intervention group received intervention in the form of a comprehensive travel health education model, while the control group received no intervention but standard health information provision.

#### Participants

The minimum sample size in this study was calculated by employing the WHO Sample Size Determination software using the formula of a 1-tailed hypothesis test for a difference in two population means [37]. Using the parameters *d*=10.0, µ1=71.2, µ2=66.2, α=.05, and 1–β= 90% and taking into account a dropout rate of 10%, the minimum number of samples for each intervention and control group was 76. Sample selection was carried out with disproportionate stratified random sampling from the list of tour guides who were HPI Bali members. The selected tour guides were contacted to determine if they met the eligibility criteria. Those who did were given a further explanation of the research and asked to sign the informed consent form if they were willing to participate.

#### Eligibility Criteria

Samples for the quantitative research were recruited according to the following inclusion criteria: registered members of HPI Bali, age 55 years old or younger, working as a tour guide for 1 year or more, own a smartphone and/or computer with Zoom and WhatsApp applications, and familiar with Zoom and WhatsApp usage.

The exclusion criteria included the following: holding structural positions in HPI, having a formal health education background, unwilling to work as a tour guide after the reopening of tourism, and reluctant to participate in the study. In addition, the dropout criteria included those who did not fully participate in the intervention and could not be contacted due to illness or other reasons at the time of data collection.

#### Assignment of Intervention

A study statistician employed permuted block randomization technique to assign the participants to the intervention and control groups [38]. The research group assignment was communicated to the research team member, who informed and managed the participants’ enrollment and intervention assignment. A strategy to improve participants’ adherence to the intervention protocol was to elucidate the benefits of full participation in this study, including the provision of a completion certificate, credit for the internet, and compensation for the time allocated for participating in the online training. In addition, adherence monitoring was performed by inspecting the participants’ attendance and participation in each education session.

#### Study Outcomes

The outcomes of this study referred to the constructs of integrated TPB and identity theory, including attitude, subjective norms, perceived behavioral control, actual behavioral control, behavioral intention, and role identity. A summary of the outcomes is presented in Table 1.

In addition to those outcome variables, several variables were controlled by analysis, namely age, sex, education, length of work, employment status, tourists’ country of origin, and type of tourism activities.

**Table 1:** Summary of study outcomes.

Study outcomes	Definition	Data measurement and analysis scale	Data source (time point)
Attitude	Psychological tendencies expressed by the tour guides regarding providing travel health information to tourists	Measurement scale: ordinal (Likert scale) Data analysis scale: interval	Self-administered questionnaire (T_0_, T_1_, T_2,_ T_3,_ T_4_)
Subjective norms	Tour guides’ perception of the extent to which providing travel health information to tourists is something that is expected by the tourists they serve, their employers (travel agents), HPI^a^, and the community	Measurement scale: ordinal (Likert scale) Data analysis scale: interval	Self-administered questionnaire (T_0_, T_1_, T_2,_ T_3,_ T_4_)
Perceived behavioral control	Tour guides' confidence in their ability to provide travel health information to tourists	Measurement scale: ordinal (Likert scale) Data analysis scale: interval	Self-administered questionnaire (T_0_, T_1_, T_2,_ T_3,_ T_4_)
Actual behavioral control	Tour guides’ knowledge regarding the prevention and first aid of travel health problems	Measurement scale: ordinal (Likert scale) Data analysis scale: interval	Self-administered questionnaire (T_0_, T_1_, T_2,_ T_3,_ T_4_)
Role identity	Tour guides’ perception of the extent to which travel health promotion is a part of their role	Measurement scale: ordinal (Likert scale) Data analysis scale: interval	Self-administered questionnaire (T_0_, T_1_, T_2,_ T_3,_ T_4_)
Behavioral intention	Tour guides’ willingness to provide travel health information to the tourists they serve	Measurement scale: ordinal (Likert scale) Data analysis scale: interval	Self-administered questionnaire (T_0_, T_1_, T_2,_ T_3,_ T_4_)

^a^HPI: Himpunan Pramuwisata Indonesia (Indonesian Tour Guide Association).

#### Data Collection

Quantitative data collection was carried out through self-administered questionnaires. The outcome variables were measured at 5 time points: at baseline (T_0_), immediately after the online training (T_1_), a month after training and before information sharing via WhatsApp (T_2_), a month after the start of WhatsApp information sharing (T_3_), and two months after WhatsApp intervention or at the end of intervention (T_4_). All participants in the intervention and control groups were asked to simultaneously fill out digital questionnaires (Google Forms) at a particular time determined by the researcher. Instructions for filling out the questionnaire were listed at the beginning of each questionnaire section to prevent incorrect or incomplete responses. Reminders via SMS and WhatsApp were sent to participants to ensure that the questionnaire was filled. There was no specific data monitoring committee established in this study due to the limited study scale and available resources. Data monitoring was undertaken by an appointed member of the research team (author NMSN).

#### Questionnaires

The instrument used to collect the quantitative data was a self-administered questionnaire. The same questionnaire was utilized for both the pretest and posttest and consisted of 7 sections: sociodemographic and job characteristics, attitude, subjective norms, perceived behavioral control, actual behavioral control, role identity, and behavioral intention.

The statements in the questionnaire to measure outcome variables were developed based on an integration of TPB and identity theory as well as the results of the qualitative strand. Alternative responses for each statement were presented on a 7-point Likert scale ranging from strongly disagree to strongly agree. Meanwhile, the actual behavioral control was measured using questions related to knowledge regarding prevention and first aid of travel-related health problems, with “right,” “wrong,” and “do not know” options.

#### Data Management and Analysis

Quantitative data were extracted from the digital questionnaires and transferred to the database. Coding was done to the extracted data, and data values were checked by two data analysts. Data without participants’ names were exported into a file format suitable for analysis with statistical software. The electronic data were stored on a computer protected with a password and only accessible to the research team.

Quantitative data will be analyzed descriptively by calculating the frequency distribution for categorical data as well as the total scores, mean, median, standard deviation, and range of minimum to maximum scores on numerical data. The comparability of the intervention group and the control group in terms of control variables will be analyzed using the chi-square test for categorical data and independent *t* test or Mann-Whitney *U* test for numerical data [39-41]. Data normality will be calculated using Kolmogorov-Smirnov test. Additionally, data homogeneity will be tested using the Levene test [39,42]. A complete case analysis will be performed to handle missing outcome data.

The mean comparison test will be conducted to determine mean differences between the intervention and the comparison groups. Repeated measures analysis of covariance (ANCOVA) will be employed because the measurement of dependent variables was conducted repeatedly at 5 time points, and there are covariates that need to be controlled in the analysis [39].

### Data Integration

Mixed methods research employs various approaches and data collection and analysis methods, which are integrated to answer a research question. Therefore, integration between the qualitative and quantitative approaches during the research process is a defining feature of mixed methods research [43]. The integration procedure in this study will be carried out through the development of joint displays that will demonstrate the relationship between quantitative and qualitative findings [32,43]. The joint display procedure aims to interpret how the results of quantitative research support the quality and the appropriateness of the context and culture of an intervention developed specifically for a particular population [32].

### Ethics Approval

This study received ethical clearance from the Institutional Review Board of the Faculty of Medicine, Udayana University/Sanglah General Hospital (number 1419/UN14.2.2.VII.14/LT/2021). The informed consent procedure was followed, and written consent was provided by the participants. Data obtained throughout the research process were kept confidential and stored in computers and cabinets only accessible to the research team. The participants’ anonymity was upheld by not including their names or other identifiable characters in transcriptions, research reports, or publications.

## Results

Qualitative data collection was conducted from May to June 2021. There were 21 informants including tour guides from 11 language divisions and the head and secretary of HPI Bali. Qualitative findings revealed some negative behavioral, normative, and control beliefs that influenced tour guides’ lack of intention and behavior. Moreover, their role as “nonhealth actors” informed their low intention to provide health information to tourists. A lack of knowledge regarding prevention and first aid of frequent travel-related health problems was also documented.

A comprehensive travel health education model was developed at the interim phase (July to October 2021). The instruments of the model, namely the training module, guidebook, and media for WhatsApp information sharing, were designed based on the qualitative findings. These instruments were further finetuned after consultation with travel medicine and health promotion experts. The questionnaire was also tested for its validity and reliability with 30 tour guides. Refinements to the questionnaire were made according to the results.

**Figure 2:**
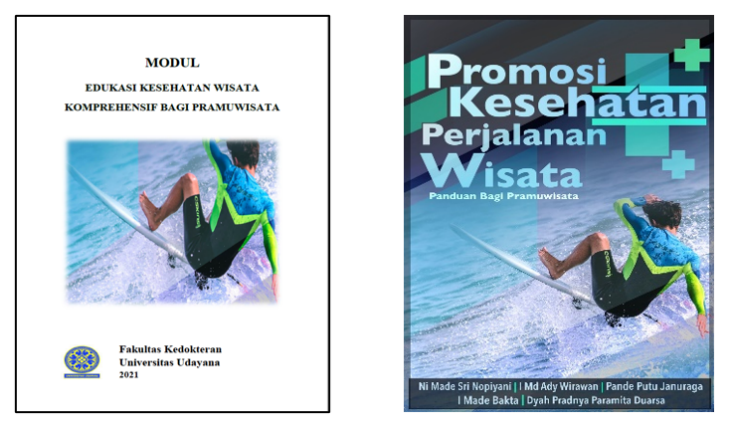
Training module and travel health guidebook.

The travel health education model includes 10 hours (2 days) of online training via Zoom and weekly travel health information sharing via the WhatsApp group for 2 months. The training content and format were included in the training module (Figure 2). The topics covered in the training included:

- The importance of travel health information for tourists
- The potential role of tour guides in tourists’ health
- Assessment of tourists’ health risks
- Prevention and first aid of general travel-related health problems, water and foodborne diseases, allergic reactions, respiratory tract infections (including COVID-19), mosquito and other insect bites, rabid animal bites, snake bites, stings and punctures from marine animals, methanol poisoning, sexually transmitted infections, and trauma or injury
- Health and safety hazards of tourist attractions and travel health facilities in Bali
- Communication techniques in travel health promotion to tourists

The training materials were delivered by travel medicine, health promotion, and medical emergency experts. Participants also received a travel health guidebook containing information on the prevention and first aid of various travel-related health problems (Figure 2).

WhatsApp information sharing was initiated in December 2021, a month after training. Participants were invited to a WhatsApp group and received weekly travel-related health information for 2 months. Participants asked questions and shared their experiences in the WhatsApp group. A moderator answered the questions and facilitated discussions among the participants. A total of 76 tour guides in the intervention group fully participated from the beginning to the end of the intervention (November 2021 to February 2022). An identical number of tour guides in the control group received no intervention but completed a series of self-administered questionnaires.

## Discussion

### Initial Findings

This study aims to develop a comprehensive, relevant, and effective travel health education model for tour guides. The model is comprehensive because it addresses all determinants of behavior according to TPB and identity theory. This model is also relevant to tour guides’ needs because it was developed with active participation from tour guides and accommodated their perceptions and preferences. The results of 21 in-depth interviews were used to develop the components of the initial travel health education model. The qualitative results of this mixed methods study confirm the findings of a cross-sectional study on tour guides in Bali showing that improvements should be made in tour guides’ provision of travel health information to tourists [23]. The qualitative data also indicate a need to address the determinants of travel health information provision to tourists, namely tour guides’ attitude, subjective norms, perceived behavioral control, role identity, actual behavioral control, and intention. The education content was developed using information on tour guides' normative beliefs, behavioral beliefs, control beliefs, role identity, and information regarding various health hazards and health problems encountered by tourists throughout their travel.

Tour guides in Bali have limited exposure to information on the prevention and first aid of travel-related health problems. Most of the informants showed a preference for training as a direct and interactive way of obtaining information regarding travel health. Due to the COVID-19 pandemic and its resultant public health measures, online training is more feasible and safer to implement than traditional face-to-face learning. Moreover, online training can be provided to tour guides residing outside Bali. Many tour guides moved back to their hometown during the pandemic because Bali is currently closed for international tourism. Training was complemented with information sharing via WhatsApp to maintain the retention of acquired information and enable further discussion. WhatsApp is considered an appropriate platform for education because it is the main communication platform used in HPI Bali.

Participation in the quantitative study was challenged by the fact that many tour guides have become jobless and are facing economic challenges due to COVID-19. Therefore, the participants were given internet credit and compensated for their time. All participants in the intervention and control groups fully participated in this study. No conclusions regarding the effectiveness of this model could be made because this study is still in the quantitative data analysis phase.

### Comparison to Prior Work

Published literature on interventions to improve the involvement of tourism actors in travel health promotion is still limited. Most of publications so far have been observational studies focusing on the travel health-related knowledge, attitude, and practices of actors in the tourism sector [23,44-47]. There was an experimental study in Canada examining the effectiveness of a health promotion model in the form of providing brochures, pamphlets, and website to improve travel agents’ referral behavior to travel clinics for pretravel consultation [31]. However, a comparison could not be made with this study due to the different target population, targeted behavior changes, and setting.

### Strengths and Limitations

The strength of this study lies in its use of an exploratory sequential mixed methods design. The qualitative strand enables a broad and in-depth exploration of the perspectives and experiences of tour guides and is expected to produce a comprehensive and relevant model. Meanwhile, the quantitative strand provides evidence of the model’s efficacy. The mixed methods design of this study can justify the efficacy, context, and cultural appropriateness of the education model developed specifically for tour guides [32].

This study has novelty as the first mixed methods research that aims to develop a travel health education model for tour guides and was developed by systematically referencing TPB integrated with identity theory [24,25]. Therefore, it is also expected to provide a theoretical advantage for the development of TPB.

This study also has some limitations. First, the education model was developed based on the experiences and perspectives of tour guides in Bali. Therefore, the content covered in this education model might not reflect the needs of all the tour guides in Indonesia because it is an archipelago with varied health situations. However, tour guides in Bali comprise more than half of tour guides in Indonesia. Tour guides in Bali, especially those proficient in languages rarely mastered by tour guides in other provinces (ie, Spanish, French, Russian, Korean, and Dutch), also provide overland tour guiding services to other parts of Indonesia. Therefore, their perceptions may also accommodate the travel health education needs of tour guides in other tourist destinations in Indonesia.

The second study limitation is that all study outcomes were measured through a self-administered questionnaire, which is a subjective measurement tool. Thus, there is a probability of participants providing normative answers that could have influenced data validity. Moreover, because this research was carried out amid the COVID-19 pandemic when tourist visits were still very limited, measurement of behavior could not be carried out.

### Future Directions

As of April 2022, this study is in the quantitative data analysis phase. The quantitative results will be integrated with the qualitative findings in a joint display to conclude on the efficacy of the intervention that was specifically designed according to the needs and context of tour guides in Bali. This study is expected to expand the amount of scientific evidence to optimize the involvement of actors in the tourism industry in travel health promotion. Therefore, at the end of this study, its findings will be disseminated through research reports, publications in journals, conferences, and public forums at national and international levels.

This study will resume once tourism in Bali has recovered and measure the effect of this intervention on tour guides' provision of health information. An investigation into the effect of tour guides’ health information to tourists, as the primary target of the intervention, will also be conducted. Some of the targeted outcomes to measure the efficacy of the intervention are tourists’ knowledge, attitude, practice, and incidence of health problems. Tourists’ perceptions and acceptance of tour guides delivering travel health advice is another aspect that warrants further investigation and will be used to develop more appropriate and effective strategies.

### Conclusions

This exploratory sequential mixed methods study aims to develop a comprehensive, relevant, and effective travel health education model for tour guides to improve their travel health promotion behavior. A travel health education model comprising online training and information sharing via WhatsApp was developed based on the qualitative findings and in consultation with experts. The intervention was trialed with tour guides, and a series of self-administrated questionnaires was completed. After the completion of quantitative data analysis, the qualitative and quantitative findings will be integrated into a joint display to conclude on the efficacy of the education model.

## Appendix

Multimedia Appendix 1 Peer-review report by the Doctoral Program of Medical Sciences, Faculty of Medicine, Udayana University (Jalan Panglima Besar Sudirman, Denpasar, Bali, Indonesia).

## Abbreviations

ANCOVA: analysis of covariance
HPI: Himpunan Pramuwisata Indonesia
TPB: theory of planned behavior
WHO: World Health Organization
