# Supplementary material for: Comprehensive Travel Health Education for Tour Guides: Protocol for an Exploratory Sequential Mixed Methods Research
Source: JMIR Res Protoc. 2022 May 23;11(5):e33840. doi: 10.2196/33840 (PMC9171602; doi:10.2196/33840)
Supplement: Multimedia Appendix 1 [file resprot_v11i5e33840_app1.pdf]

KEMENTERIAN PENDIDIKAN DAN KEBUDAYAAN
UNIVERSITAS UDAYANA
FAKULTAS KEDOKTERAN
PROGRAM STUDI DOKTOR ILMU KEDOKTERAN
Jalan Panglima Besar Sudirman Denpasar Bali
Telp/Fax: 0361-223797/(0361)-247962 Ext: 115/204, Laman : www.unud.ac.id

**List of inputs/comments and revision**		
Name	:	Ni Made Sri Nopiyani, MD., MPH
Student ID	:	1990211019
Dissertation tittle	:	Development of A Comprehensive Travel Health Education Model to Improve Tour Guides' Attitude, Subjective Norm, Perceived Behavioral Control, Role Identity, Actual Behavioral Control, and Intention to Provide Travel Health Information to Tourists in Bali
Date of examination	:	5th of February 2021

**NO**	**Examiners**	**Inputs/Comments**	**Revision**
1	Prof. Ari Natalia Probandari, MPH, Ph.D<br><br>(Approved the revision in 2 March 2021)	1. Specific objective number 1 should be changed into a more relevant objective for a qualitative study, for example: "to explore stakeholder views on a comprehensive and relevant travel health education model to the needs of tourists."<br>2. Change the verb used in the subsequent specific objectives: 'to know' to 'to prove'<br>3. Add hypothesis for the qualitative study<br>4. In the method section: add the development of joint displays at the end of the study to link the results of qualitative and quantitative research.<br>5. In-depth interview guidelines need to be incorporated with questions that further explore the educational methods	1. Specific objective number 1 has been revised.<br>2. Verbs in the subsequent objectives have been revised.<br>3. Hypothesis for qualitative study has been added.<br>4. Development of joint display after the quantitative analysis has been added.<br>5. Interview guidelines have been revised.<br>6. Term 'intervention model' has been replaced with 'education model'<br>7. Education model has been described in more detail.<br>8. Typo has been fixed.<br>9. Long sentences have been shortened.

NO	Examiners	Inputs/Comments	Revision
		(who gives it, where it is given, etc.). The questions in the interview guide are also too general that it is at risk of producing normative answers. Change the sentence or add probing below the questions.<br>6. Consistent use of term for educational model throughout the protocol.<br>7. The education model should be described in more detail which include who provides information, who are the target audience, what are the methods of information provision, what is the educational content, what is the language used in the education.<br>8. Fix the typo<br>9. Break long sentences into shorter and more concise sentences.<br>10. Abbreviations must be preceded by the full form<br>11. Terms in foreign languages should be written in italic	10. Full form has been added prior to its abbreviation.<br>11. Terms in foreign language have been written in Italic.
2	Prof. Dr. dr. I Made Jawi, M.Kes, Dr.PH<br><br>(Approved the revision on 22 February 2021)	1. State the main problem more precisely in the introduction (whether the problem is lack of knowledge or behavior or other?) And explain how this education model will address the existing problems.<br>2. Explain the advantages of the educational model developed in this study over the standard information provision.<br>3. Explain more about how to reduce bias in the measurement with a questionnaire.	1. The problem has been explained in the background.<br>2. The advantages of education model over the standard information provision have been explained.<br>3. Validity and reliability tests of the questionnaire have been explained in the methods section.
3	Prof. Dr. dr. Nyoman Mangku Karmaya, M.Repro, PA(K)	1. In the research concept, make intention as the dependent variable and the other outcome variables as intermediate variables.	1. Intention has been made as dependent variable and other outcome variables have been

NO	Examiners	Inputs/Comments	Revision
		2. Background: how to determine high, medium, low risk tourist areas?<br>3. Things to consider: what do tourists think about the provision of health information by tour guides? Is it acceptable for them?<br>4. Describe in more detail of what comprised comprehensive travel health education model and the standard tourist health information provision.	made as intermediate variables.<br>2. The risk is determined using WHO criteria (has been explained in background).<br>3. The question has been accommodated in the interview guide.<br>4. The tentative model has been described.
4	Dr. dr. Dewa Made Sukrama, M.Si, Sp.MK (K)<br><br>(Approved the revision on 22 February 2021)	1. Explain further about how the output of the research process produces novelty.<br>2. How the data collection (interviews) will be conducted? Pay attention to the interview method due to the risk of COVID-19.<br>3. Age of research subjects should be made more homogeneous because it can affect the results of the intervention.	1. It has been explained in the novelty section.<br>2. It has been explained in the qualitative method section.<br>3. Age has been made as one of the eligibility criteria.
5	Dr. dr. Dyah Pradnyaparamita Duarsa, M.Si<br><br>(Approved the revision on 20 February 2021)	1. External factors such as Indonesian Tour Guides Association (HPI) or travel agents' policies need to be considered.<br>2. In the preparation of the educational model there may be a role model culture so that education can be in the form of Training of Trainer (TOT).<br>3. Research's practical advantage: Research results can be advocated to HPI leaders so that tourism health education models can be integrated.<br>4. Fix typo.	1. Employer's policy becomes the variable under study (control variable). The HPI's policy is not investigated because it has the same effect on subjects in the intervention and comparison groups.<br>2. It has been added to the questions in the interview guide.<br>3. It has been added in the research's practical benefits.<br>4. Typo has been fixed

KEMENTERIAN PENDIDIKAN DAN KEBUDAYAAN
UNIVERSITAS UDAYANA
FAKULTAS KEDOKTERAN
PROGRAM STUDI DOKTOR ILMU KEDOKTERAN
Alamat: Jl. PB Sudirman Denpasar Bali. 8022[illegible]
Telepon: (0361) 222510 Fax: (0361) 246656
Laman: www.unud.ac.id

TO WHOM IT MAY CONCERN

This letter is to certify that:

Name : Ni Made Sri Nopiyani, MD, MPH
Position : Doctoral student
Institution : Doctoral of Medical Sciences Study Program, Faculty of Medicine, Udayana University.

Has been granted Domestic Post Graduate Scholarship by The Indonesian Ministry of Education and Culture from August 2019 to August 2022.
The Scholarship provides financial support for the doctoral education including the implementation of the research.

Denpasar, 26 July 2021
Head of Medical Sciences Study Program
Faculty of Medicine, Udayana University

Prof. Dr. dr. I Made Jawi, M.Kes
